# Early economic modeling of magnetic resonance image-guided high intensity focused ultrasound compared to radiotherapy for pain palliation of bone metastases

**DOI:** 10.3389/fonc.2022.987546

**Published:** 2022-09-23

**Authors:** Julia Simões Corrêa Galendi, Sin Yuin Yeo, Holger Grüll, Grischa Bratke, Dennis Akuamoa-Boateng, Christian Baues, Clemens Bos, Helena M. Verkooijen, Arim Shukri, Stephanie Stock, Dirk Müller

**Affiliations:** ^1^ Institute of Health Economics and Clinical Epidemiology, Faculty of Medicine and University Hospital of Cologne, University of Cologne, Cologne, Germany; ^2^ Institute of Diagnostic and Interventional Radiology, Faculty of Medicine and University Hospital of Cologne, University of Cologne, Cologne, Germany; ^3^ Department of Chemistry, Faculty of Mathematics and Natural Sciences, University of Cologne, Cologne, Germany; ^4^ Department of Radiation Oncology, CyberKnife and Radiotherapy, Faculty of Medicine and University Hospital Cologne, University Hospital of Cologne, Cologne, Germany; ^5^ Division of Imaging and Oncology, University Medical Center Utrecht, Utrecht University, Utrecht, Netherlands

**Keywords:** bone metastases, pain palliation, cancer pain, cost-effectiveness, high-intensity focused ultrasound, MR-HIFU, radiotherapy

## Abstract

**Introduction:**

Magnetic Resonance Image-guided High Intensity Focused Ultrasound (MR-HIFU) is a non-invasive treatment option for palliative patients with painful bone metastases. Early evidence suggests that MR-HIFU is associated with similar overall treatment response, but more rapid pain palliation compared to external beam radiotherapy (EBRT). This modelling study aimed to assess the cost-effectiveness of MR-HIFU as an alternative treatment option for painful bone metastases from the perspective of the German Statutory Health Insurance (SHI).

**Materials and methods:**

A microsimulation model with lifelong time horizon and one-month cycle length was developed. To calculate the incremental cost-effectiveness ratio (ICER), strategy A (MR-HIFU as first-line treatment or as retreatment option in case of persistent pain or only partial pain relief after EBRT) was compared to strategy B (EBRT alone) for patients with bone metastases due to breast, prostate, or lung cancer. Input parameters used for the model were extracted from the literature. Results were expressed as EUR per quality-adjusted life years (QALYs) and EUR per pain response (i.e., months spent with complete or partial pain response). Deterministic and probabilistic sensitivity analyses (PSA) were performed to test the robustness of results, and a value of information analysis was conducted.

**Results:**

Compared to strategy B, strategy A resulted in additional costs (EUR 399) and benefits (0.02 QALYs and 0.95 months with pain response). In the base case, the resulting ICERs (strategy A vs. strategy B) are EUR 19,845/QALY and EUR 421 per pain response. Offering all patients MR-HIFU as first-line treatment would increase the ICER by 50% (31,048 EUR/QALY). PSA showed that at a (hypothetical) willingness to pay of EUR 20,000/QALY, the probability of MR-HIFU being cost-effective was 52%. The expected value of perfect information (EVPI) for the benefit population in Germany is approximately EUR 190 Mio.

**Conclusion:**

Although there is considerable uncertainty, the results demonstrate that introducing MR-HIFU as a treatment alternative for painful bone metastases might be cost-effective for the German SHI. The high EVPI indicate that further studies to reduce uncertainty would be worthwhile.

## Introduction

Bone metastases occur in 65% of patients with advanced solid cancer, particularly originating from malignancies of the lung, prostate, and breast. For these patients, pain is a common and devastating symptom affecting both quality of life and functionality (1–3). Opioids are regularly the baseline pharmacologic treatment for pain palliation. However, high doses required to manage pain effectively are associated with numerous adverse effects (2). Since patients with persistent pain often require additional focal treatment, loco regional external beam radiotherapy (EBRT) is the current standard of care for patients with bone metastases (1, 4, 5).

Approximately 60-70% of patients initially respond to EBRT over the course of four weeks following treatment (6–8). However, among those adequately responding to EBRT, about 50% experience recurrent pain (9). For those non-responding to EBRT or suffering recurrent bone pain, re-irradiation is limited as cumulative radiation doses might be harmful for organs at risk surrounding the target lesion. In addition, only 58% of patients undergoing re-irradiation benefit from it (9).

Magnetic Resonance Image-guided High Intensity Focused Ultrasound (MR-HIFU) is a non-invasive treatment modality that may substantially improve pain palliation and can be offered as first-line treatment or after prior radiation (5). A randomized placebo-controlled trial demonstrated that MR-HIFU is superior to placebo after 3 months: the response rate for the primary endpoint (improvement in self-reported pain) was 64% in the MR-HIFU arm compared to 20% in the placebo arm (P <.001) (10). Although to date there is no randomized controlled trial (RCT) comparing MR-HIFU with EBRT directly, a single-center matched-pair study showed similar overall treatment response rates but faster pain relief using MR-HIFU compared to EBRT (pain relief in 71% vs. 26% at 1 week, p = 0.0009 and 81% vs. 67%, p = 0.3753 at 1 month) (11). Moreover, MR-HIFU has less side effects (5).

An early assessment of the cost-effectiveness of adding MR-HIFU as first-line treatment or after prior radiation compared to EBRT can provide an appraisal of the potential value of this new technology (e.g., to support reimbursement decisions, investment in installation of medical infrastructure and research prioritization). This economic modelling study assessed the cost-effectiveness of MR-HIFU as treatment alternative for the palliative treatment of patients with bone metastases in comparison to the current standard of care (i.e., EBRT alone), from the perspective of the Statutory Health Insurance (SHI) in Germany.

## Material and methods

To reflect the clinical and economic consequences of MR-HIFU and EBRT for the treatment of bone metastases, we developed a patient-level simulation model (software TreeAge Pro 2019) with a lifetime horizon and a one-month cycle length. The cycle length was chosen because retreatment of patients with painful bone metastases can be considered after one month of persistent pain (12). The analysis was performed from the perspective of the SHI which covers 87% of the German population (13).

Patients entering the model were assumed to be male and female adults with non-vertebral painful bone metastases originating from lung cancer, prostate cancer, or breast cancer in an even distribution. The model population reflected that over 80% of bone metastases from solid tumors arise from cancers of the breast, prostate, or lung (14). In the model, patients were referred to treatment with MR-HIFU or EBRT due to significant pain (scoring at least four by the Numerical Rating Scale, NRS), having received optimal pain management with opioids.

### Strategies for the comparison

**Figure 1:**
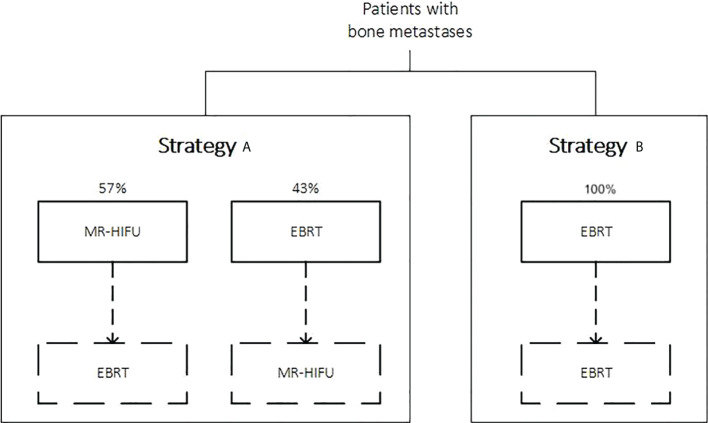
Strategies being compared. Dashed lines refer to the possibility of a retreatment in case of persistent pain or partial pain relief after a first-line treatment (i.e., not all patients will undergo a retreatment in their lifetime, since some patients might die, or remain with unpalliated pain for some time before being recommended a retreatment).

For the main comparison, two strategies were outlined. Strategy A was defined as MR-HIFU either as a first-line treatment (about 60%) or as retreatment option after failed EBRT (about 40%). In strategy A, not all patients received MR-HIFU as first line-treatment because in a realistic scenario MR-HIFU is unlikely to replace EBRT completely as first-line treatment. Patients who receive EBRT as a first-line treatment were assumed to be re-treated with MR-HIFU in case of persistent pain or only partial pain relief. The proportions were chosen to confer more internal consistency with the trial informing data on MR-HIFU effectiveness (10). Strategy B reflected the standard of care practice in Germany, defined as EBRT followed by re-irradiation in case of persistent pain. The EBRT dose was mainly multi-fraction (i.e., 20Gy in five daily fractions), and single-fraction (8Gy in one fraction) for 10% of cases, reflecting the preferred practices in German radiotherapy institutions (15, 16), and recommendations for treatment of patients with more favorable prognosis (i.e., life expectancy more than four weeks) from the German guideline (17). **Figure 1** shows the strategies for the comparison.

### Model overview

**Figure 2:**
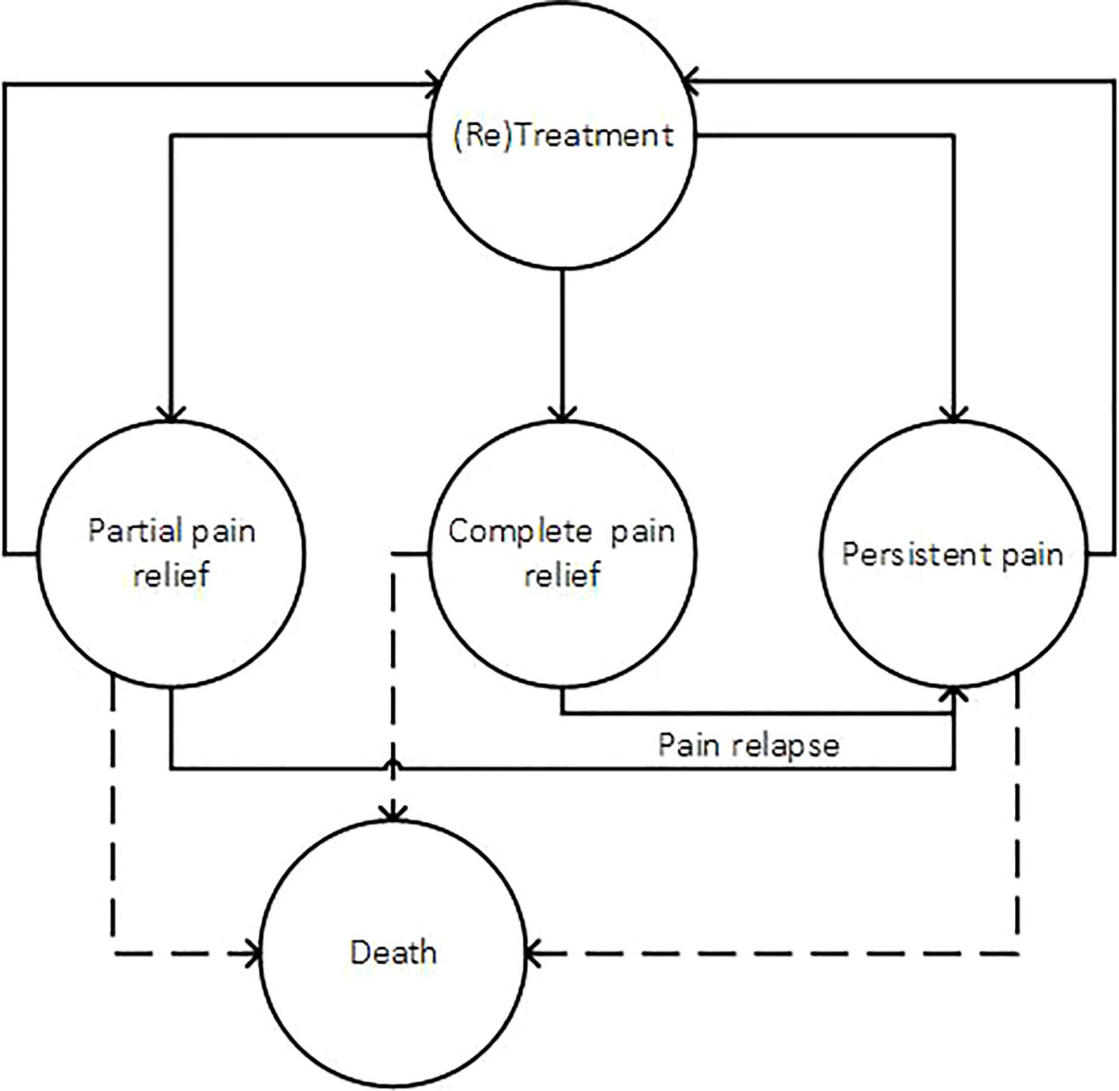
Model overview. Patients enter the model after treatment with either EBRT or MR-HIFU. Pathological fracture was modelled as an event that could occur in each cycle and health state (except death).

The patient-level simulation model reflected the clinical course that may follow palliative treatments with MR-HIFU or EBRT: i. complete pain relief (pain score of zero in the NRS), ii. partial pain relief (i.e., defined as a reduction of pain score of at least two points without increase of analgesic intake), iii. persistent pain, iv. retreatment in case of persistent pain or pain relapse and v. death. In addition, the risk of suffering a pathological fracture was considered as an event that could occur in any health state except death, because of its economic consequences and potential impact on quality of life. **Figure 2** shows the model overview.

### Input parameters

Several systematic literature searches in Medline (*via* PubMed) were performed to identify adequate input parameters (e.g., event probabilities, utilities, and costs). Studies were selected with regard to methodological quality and representativeness for the German context. Input parameters are reported in **Table 1**.

**Table 1:** Model input parameters.

		
Input parameter	Value	Source
Event probabilities	Monthly probability (SD)	
MR-HIFU		
Complete response (1 week after treatment)	0.230 (0.04)	(10)
Partial response (1 week after treatment)	0.410 (0.04)	(10)
No response (1 week after treatment)	0.350 (0.04)	(10)
Retreatment	0.018 (0.0016*)	Assumption (18),
Pathological fracture	0.003 (0.005)	(10)
Multi-fraction EBRT		
Complete response (4 weeks after treatment)	0.240 (0.008)	(18)
Partial response (4 weeks after treatment)	0.380 (0.008)	(18)
No response (4 weeks after treatment)	0.380 (0.008)	(18)
Retreatment	0.007 (0.0011)	(18)
No response after retreatment	0.420 (0.021)	(9, 18)
Pathological fracture	0.003 (0.0007)	(18)
Single-fraction EBRT		
Complete response (4 weeks after teatment)	0.230 (0.008)	(18)
Partial response (4 weeks after treatment)	0.380 (0.008)	(18)
No response (4 weeks after treatment)	0.390 (0,008)	(18)
Retreatment	0.018 (0.0016)	(18)
No response after retreatment	0.420 (0.021)	(9, 18)
Pathological fracture	0.003 (0.0007)	(18)
Pain relapse in both strategies	0.022 (0.008)	(19)
Monthly probability of death after bone metastasis diagnosis		
Breast cancer	1y: 0.040 (0.0004); 2y: 0.029; 3y: 0.029; 4y: 0.027; 5y: 0.027	(14, 20)
Prostate cancer	1y: 0.053 (0.0018); 2y: 0.039; 3y: 0.034; 4y: 0.029; 5y: 0.028	(14)
Lung cancer	1y: 0.070 (0.0005); 2y: 0.050; 3y: 0.050; 4y: 0.030; 5y: 0.020	(14)
Health state utilities	QALYs adjusted for 1-month cycle (SD)	
Basic health state (painful bone metastases)	0.039 (0.035)	(21)
Pathological fracture	- 0.009 (0.021)	(21)
Multi-fraction EBRT	- 0.009 (0.025)	(21)
Single-fraction EBRT	- 0.004 (0,014)	(21)
MR-HIFU	- 0.005 (0.014)	Assumption (21),
Complete pain relief	+ 0.019 (0.001)	(22)
Partial pain relief	+ 0.008 (0.001)	(22)
Costs	Value in EUR (SD)	
MR-HIFU		
Out-patient diagnostic MRI	118	(23)
In-patient treatment (gDRG)*	3,430	(24)
MR-HIFU, cost-covering lump-sum (best- and worst-case scenarios)	5,147 (4,092 – 5,876)	(25)
Multi-fraction EBRT		
Out-patient treatment*	2,411	(26)
In-patient treatment (gDRG)*	6,410	(24)
Proportion EBRT out-patient*	70%	Expert opinion (15, 16, 27),
Single-fraction EBRT	1486	(24)
Proportion of 1x 8Gy EBRT	10%	(15, 16, 27)
Pathological fracture (total)	21,430 (8572)	(23)
Out-patient	1,593 (637)	(23)
In-patient	12,596 (5038)	(23)
Rehabilitation	203 (81)	(23)
Out-patient prescriptions	5,446 (2178)	(23)
Aid and remedies	1,592 (637)	(23)
Oxycodone 20mg each 4 hours (monthly costs)	210 (84)	(28)

*Standard deviation assumed to be 20% of mean value. EBRT, External Beam Radiation Therapy; MR-HIFU, Magnetic Resonance-guided High Intensity Focused Ultrasound; MRI, Magnetic Resonance Imaging; QALY, Quality-adjusted Life Years; SD, Standard Deviation.

#### Event probabilities

Data on effectiveness of EBRT on inducing complete or partial pain palliation and risk of pathological fracture were extracted from a recently published systematic review of RCTs comparing single-fraction and multi-fraction-EBRT (18). The effectiveness of MR-HIFU for complete or partial pain relief was extracted from a RCT including 112 patients with a three-month follow up (10). Effectiveness of MR-HIFU in case of upstream EBRT (strategy A) was assumed to be the same as for MR-HIFU offered as first-line treatment. Effectiveness of retreatment with the EBRT for achieving complete or partial pain relief (strategy B) was slightly inferior because there is some evidence that re-irradiation is less effective than EBRT for radiation-naive patients (9).

Probability of pain relapse with EBRT was taken from the Bone Pain Working Party Trial, a RCT comparing multi-fraction versus single-fraction EBRT (19). This study was chosen because over 98% of patients in the multi-fraction arm were treated with a fractionation scheme similar to that used for our model (20Gy in 5 fractions). In this cohort, the one-year-cumulative probability of a pain relapse was 30% (19). Because of a lack of evidence for the probability of a pain relapse (resulting in retreatment after first-line treatment with MR-HIFU), we assumed equal rates of pain relapse for MR-HIFU and EBRT, considering that recurrence of pain is mainly driven by progression of the disease (19, 29).

In the literature, annual retreatment rates after multi-fraction EBRT are reported at 8%, and that after single-fraction 20% (2.5 times higher than for multi-fraction EBRT) (18). The retreatment rate after multi-fraction EBRT is lower, probably due to concerns with the cumulative radiation dose of multi-fraction EBRT, even though the time to pain increase is similar in single-fraction and multi-fraction EBRT (6, 19). Since the retreatment rate related to MR-HIFU is unknown, in strategy A we applied the retreatment rate of single-fraction EBRT (i.e., 20% annually). The uncertainty of this assumption was tested in sensitivity analyses considering a range of retreatment rates for MR-HIFU.

Cancer-specific overall survival (OS) was obtained from a Danish population-based cohort study that included 17,251 patients with bone metastases (14). In that study, one-year and five-year OS after diagnosis of bone metastases for patients with prostate cancer were 35% and 6%, respectively, while patients with lung cancer had a 10% one-year OS and a 1% five-year OS (14). The OS of patients with metastasized breast cancer in that study was in line with a prospective multicenter cohort study of German patients with breast cancer metastasized to the bone (i.e., five-year OS of 22%) (20).

#### Utilities (Quality-adjusted life years)

Health state utility values were taken from a time trade-off study from Matza et al, which elicited utility values for patients with bone metastases and skeletal-related events (i.e., fractures and radiation to the bone) from 187 participants living in the United Kingdom (UK) and Canada (21). The increase in utility due to partial and complete pain relief were taken from a study that elicited utilities for different intensities of chronic pain (22). The increases in utility due to complete/partial pain relief were 35% and 15% from the base state, respectively. Increases in utilities due to complete or partial pain relief were assumed to occur within seven days after MR-HIFU, and within four weeks after EBRT (30).

Utilities were subtracted due to adverse events related to treatment/retreatment and pathological fractures. Common adverse events associated with EBRT are nausea and vomiting for two weeks following treatment (19). Reported adverse events associated with MR-HIFU are discomfort or pain due to positioning, fatigue or numbness that resolve within one day after treatment (10). Decreases in utilities reported for single-fraction and multi-fraction EBRT were 0.05 and 0.11, respectively (21). For MR-HIFU, data on utility has not yet been published. Hence, the utility of MR-HIFU was assumed an average of single/multi-fraction EBRT (0.07 QALY). This assumption was based on expert opinion, considering that MR-HIFU is associated with reduced hospital time and adverse effects than multi-fraction EBRT. Compared to single-fraction EBRT, however, MR-HIFU requires general anesthesia and overnight stay which may be burdensome for patients.

#### Costs

Costs of MR-HIFU included one overnight stay at the hospital, general anesthesia and one post-treatment MRI. An additional pre-treatment out-patient MRI was considered in case MR-HIFU was performed as first-line treatment. Depending on the general condition of the patient and the total dose required, in Germany, EBRT is performed as in- or out-patient treatment. Published cost-of-illness studies and surveys indicate that the proportion of out-patient treatments in Germany is 50-60% (15, 16, 23, 27) with no significant difference between German general hospitals, practices, and university hospitals (15). However, these studies assessed bone metastases in general (including complicated bone metastases, patients receiving post-operative radiation for spinal metastases, sometimes with pronounced neurological symptoms), while our patient population (uncomplicated bone metastases) is less likely to require in-patient treatment. Hence, for the base case, we assumed the proportion of out-patient EBRT to be 70% for the base case.

According to the perspective of the SHI, direct medical costs related to EBRT and MR-HIFU were based on the German Physicians’ Fee Schedule 2022 (Einheitlicher Bewertungsmaßtab) for out-patient procedures (26), and the 2022 German diagnosis related group (gDRG) weights (for in-patient procedures) (24). The diagnosis and procedure codes considered for the cost calculations are detailed on the **Supplementary Material (SM1)**.

In line with similar models, for all health states except for complete pain relief, costs with pain medication were estimated considering oral oxycodone as a reference medication (29, 31). For patients with persistent pain and partial pain relief, an intake of oral oxycodone 20 mg every four hours was assumed (29, 31). For the pricing of pain medication, we referred to the German formulary 2022 (28). Costs of bone targeting agents to prevent fractures (e.g., bisphosphonates) were not included because they would impact both treatment strategies equally.

Costs associated with a pathological fracture were extracted from a retrospective cost-of-illness study based on German claims data including 2434 patients with bone metastases and solid tumours (23). These costs included in- and out-patient consultations, rehabilitation, out-patient prescriptions, aids, and remedies (23). Costs were adjusted for inflation to the target year 2021 based on the harmonized index of consumer price (32).

### Model outputs

To compare the alternatives, the incremental cost-effectiveness ratio (ICER) was calculated as cost per pain response (i.e., months spent in complete or partial pain response) and cost per QALY. Because survival after diagnosis of bone metastasis varies by cancer type, in subgroup analyses, the ICER was calculated for each primary cancer diagnosis (i.e., breast, prostate, and lung cancer). Costs and benefits were discounted at a 3% annual rate (33).

### Model validation and sensitivity analyses

To validate the model, we consulted experts on the adequacy of input data and the conceptual appropriateness of the model. Technical accuracy was checked regarding data entry and programming errors. For cross model validation, we compared our assumptions to those in similar models. We report the validation efforts in detail in the **Supplementary Material (SM2)**, following the ‘Assessment of the Validation Status of Health Economic decision models’ checklist (34).

In deterministic sensitivity analyses (DSA) all input parameters were varied, except for the cancer-specific mortality rates. Structural sensitivity analyses were performed to calculate the ICER considering different scenarios: i. all patients receiving MR-HIFU as first-line treatment in strategy A, ii. alternative retreatment rates in strategy A (e.g., same retreatment rates as multi-fraction EBRT and double that of single-fraction EBRT), iii. a cost-covering lump-sum for MR-HIFU, iv. a range of proportions of single-fraction EBRT (in both strategies), v. a range of proportions for out-patient EBRT (in both strategies). The cost-covering lump sum was taken from a recent time-driven activity-based costing study prospectively conducted at an university hospital from the hospital perspective (25). The cost-covering lump sum includes capital costs for MR-HIFU equipment, which are not incorporated in the calculation of gDRG lump sums (25, 33).

A probabilistic sensitivity analysis (PSA) was conducted to test the robustness of the results. Because there is no commonly accepted willingness-to-pay threshold for Germany, the probability of strategy A being cost-effective was assessed for different levels of willingness-to-pay (WTP) (i.e., hypothetical thresholds, at which the SHI would accept the additional costs for an additional benefit) (35).

### Value of information (VOI) analysis

A VOI analysis was conducted to estimate the value of collecting additional evidence (e.g., a RCT comparing MR-HIFU with EBRT) for reducing uncertainty of the analysis (36). While the expected value of perfect information (EVPI) indicates whether the cost of conducting new research is worthwhile (i.e. should we collect more evidence)? (36), the expected value of perfect partial information (EVPPI) quantifies how individual parameters or parameters sets contribute to decision uncertainty (i.e., what evidence should we collect)? (36). The EVPI and the EVPPI were calculated using the Sheffield Accelerated Value of Information (SAVI) tool (37), and epidemiologic data from the German Centre for Cancer Registry Data (38). More information is provided in the **Supplementary Material (SM3)**.

## Results

### Base case results

Compared to strategy B (EBRT alone), strategy A (with MR-HIFU) resulted in slightly higher costs (EUR 399) and more benefits (0.02 QALYs and 0.95 months with pain response), with ICERs of EUR 19,845 per QALY and EUR 421 per month with pain response. Limiting the analysis to cancer-subgroups, strategy A resulted in increased costs and more benefits (breast cancer: 22,403 EUR/QALY and 484 EUR/pain response, prostate cancer: 21,072 EUR/QALY and 2,281 EUR/pain response, and lung cancer: 14,086 EUR/QALY and 188 EUR/pain response). **Table 2** shows the results for the base case.

**Table 2:** Base case results and subgroup analyses according to primary diagnosis.

	Cost	Incremental cost	Effectiveness		Incremental effectiveness		ICER	
	EUR	EUR	QALY	Pain response	QALY	Pain response	EUR/QALY	EUR/pain response
**Base case**								
Strategy B	8115	–	0.94	9.41	–	–	–	–
Strategy A	8514	399	0.96	10.36	0.020	0.95	19,845	421
**Breast cancer**								
Strategy B	9401	–	1.15	11.16	–	–	–	–
Strategy A	9852	451	1.17	12.40	0.027	1.23	22,403	484
**Prostate cancer**								
Strategy B	8609	–	0.95	9.64	–	–	–	–
Strategy A	8241	368	0.97	10.55	0.018	0.91	21,072	2,281
**Lung cancer**								
Strategy B	7417	–	0.73	7.48	–	–	–	–
Strategy A	7227	190	0.74	8.19	0.015	0.71	14,086	1,592

Strategy B, EBRT alone; strategy A, with MR-HIFU. QALY, Quality-adjusted life-years gained; ICER, Incremental cost-effectiveness ratio; MR-HIFU, Magnetic resonance-guided High Intensity Focused Ultrasound. Pain response defined as months spent with palliated pain.

### DSA and structural sensitivity analyses

In DSA, the variables with the highest impact on the ICER were the effectiveness of MR-HIFU for complete pain relief, the retreatment rate in strategy A, the MR-HIFU treatment costs, and EBRT costs in that order. In the **Supplementary Material (SM4)**, results of DSA are shown in a tornado diagram (**Figure S1**).

In structural sensitivity analyses, applying alternative retreatment rates in strategy A resulted in similar results as in the base case (i.e., strategy A costs more and generates more QALY). Higher retreatment rates in strategy A resulted in higher ICERs, meaning that the more often retreatments were performed, the lesser cost-effective strategy A was. Moreover, offering MR-HIFU as first-line treatment to all patients at strategy A resulted in higher additional costs (EUR 721 vs. 364 in the base case) and slightly more QALYs (0.023 vs. 0.020 in the base case). The resulting ICER in this scenario (31,048 EUR/QALY) is 50% increased compared to the base case. Furthermore, structural sensitivity analyses assuming less costly EBRT practices (i.e., higher proportions of 1x8Gy dose or out-patient treatment) resulted in higher ICERs (i.e., strategy A is less likely cost-effective). Complete results are provided in **Table S6** (in **SM4**) (**Table 3**).

**Table 3:** Structural sensitivity analyses results.

	Cost	Incremental cost	Effectiveness	Incremental effectiveness	ICER
	EUR	EUR	QALY	QALY	EUR/QALY
Retreatment rate at Strategy A defined at 8% (same as MF-EBRT)					
Strategy B	8,115	–	0.94	–	–
Strategy A	8,500	385	0.96	0.02	18,531
Retreatment rate at Strategy A defined at 32% (4-fold MF-EBRT, 2-fold the base case)					
Strategy B	8,115	–	0.94	–	–
Strategy A	10,106	1,991	0.99	0.05	38,808
All patients receiving MR-HIFU as first-line treatment (at Strategy A)					
Strategy B	8,115	–	0.94	–	–
Strategy A	8,836	721	0.96	0.02	31,048
Cost-covering lump-sum MR-HIFU					
Strategy B	8115	–	0.94	–	–
Strategy A	9663	1548	0.96	0.02	77,650
All EBRT dose 1x 8Gy (at both strategies)						
Strategy B	6,214	–	0.95	–	–
Strategy A	7,388	1,174	0.96	0.01	168,392
All EBRT as out-patient treatment (at both strategies)						
Strategy B	6,604	–	0.94	–	–
Strategy A	7,742	1,138	0.96	0.02	56,566

Strategy B, EBRT alone; strategy A, with MR-HIFU. Abbreviations. QALY, Quality-adjusted life-years gained; ICER, Incremental cost-effectiveness ratio; MR-HIFU, Magnetic resonance-guided High Intensity Focused Ultrasound, MF-EBRT, Multi-fraction External Beam Radiotherapy.

### PSA

**Figure 3:**
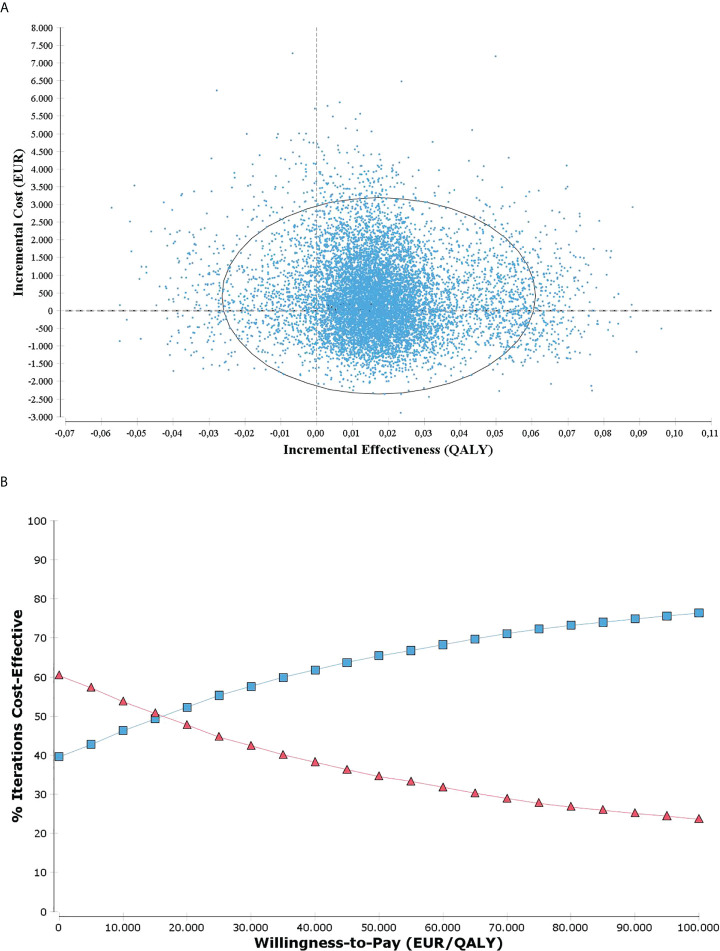
**(A)** Incremental cost-effectiveness plane with 10.000 iterations resulting from probabilistic sensitivity analysis with 95% confidence ellipse; **(B)** Cost-effectiveness acceptability curve for a range of willingness-to-pay values. Strategy A indicated in blue and strategy B in red.

In PSA, the iterations spread across the four quadrants of the cost-effectiveness plane (**Figure 3A**). Fifty-three percent of the iterations fall into the upper right quadrant, corresponding to the base case result (i.e., strategy A resulted in more costs and more QALYs), while 36% of the iterations fall into the lower right quadrant, indicating that strategy A may result in more QALYs and be cost saving. However, in 10% of the iterations strategy A was less effective than strategy B (upper and lower left quadrants). As a result, at a WTP of EUR 20,000/QALY, the probability of strategy A being cost-effective is 52% (i.e., in 48% of the iterations the additional costs per QALY were above the (hypothetical) threshold of EUR 20,000). At a WTP of EUR 40,000/QALY, the probability of strategy A being cost-effective is 64% and at EUR 60,000/QALY, 69%. **Figure 3B** shows the cost-effectiveness acceptability curve for a range of willingness-to-pay values.

### VOI analysis

The EVPI for the choice between strategy A and strategy B was EUR 434 per person affected by the decision. Extrapolated to the German population over a period of five years, the EVPI was EUR 178 Mio. These values represent the cost of making the decision based on current (uncertain) evidence and set the maximum amount that should be applied into additional research to reduce uncertainty of the analysis. The parameters with the highest EVPPI per person were MR-HIFU costs (EUR 329, SD:5) and the fracture rate following MR-HIFU (EUR 151; SD: 8). Further relevant parameter sets worthy of collecting further information were: QALY values (EUR 67, SD: 4), proportion of single-fraction and of out-patient EBRT jointly (EU 62, SD: 6) and effectiveness of MR-HIFU on pain palliation (EUR 53, SD: 6), as detailed on the **Supplementary Material (SM5)**.

## Discussion

In comparison to EBRT, the MR-HIFU strategy resulted in higher costs and more benefits (QALYs and months of pain response) for patients with bone metastases. The overall results were confirmed in subgroup analyses for breast cancer, prostate cancer, and lung cancer. Patients with bone metastases due to lung cancer had the lowest lifetime (cumulative) costs and benefits, probably because 90% of these patients died within the first year. Because the added benefit of MR-HIFU is short-term (i.e., faster pain relief than EBRT) and the most impactful additional costs are long-term (i.e., costs of retreatment and fracture), strategy A was more cost-effective for this subgroup of patients with poorer prognosis. While the German SHI cannot expect savings with MR-HIFU, the cost-effectiveness was similar to various medical interventions for bone metastases (39).

Due to a lack of appropriate data (e.g., a direct clinical comparison between the treatment alternatives) the model had to be based on different clinical studies several assumptions. As a result, at a WTP of 20,000 EUR/QALY, the probability of MR-HIFU being cost-effective is 52%, whereas for a WTP of 60,000 EUR/QALY the probability is 69%. In Germany, there is not a commonly accepted WTP threshold to determine reimbursement decisions. For WTP thresholds higher than 20,000 EUR/QALY, the potential cost-effectiveness might justify investments in infrastructure installation. Moreover, early economic models are useful to explore (i) MR-HIFU’s role in the clinical management of bone metastases and (ii) the potential value of further research (40, 41).

Because the role of MR-HIFU in the clinical management of painful bone metastases is still incipient, we explored several alternative scenarios in structural sensitivity analyses. For example, it was detectable that higher retreatment rates at strategy A tend to increase the ICER. Furthermore, a scenario with MR-HIFU being offered as first-line treatment for all patients increased the ICER (31,048 EUR/QALY), due to a higher increase in costs despite a slight increase in QALYs. Although not considered in our calculations, some case series indicate that patients without prior radiation might respond better to MR-HIFU than those with prior radiation (30, 42). The mechanism of action supporting this finding warrants further investigation. If this early evidence from case series is confirmed in larger samples, the cost-effectiveness of MR-HIFU as first-line treatment would be improved.

Repeated irradiations from EBRT are limited due to normal tissue tolerance (6, 19). In contrast, MR-HIFU could be repeated for non-responders since there is theoretically no limit for the accrued acoustic energy (5, 30). However, the possibility of repeating MR-HIFU (i.e., MR-HIFU after initial treatment with MR-HIFU) was not considered in this model, because to date there is not sufficient clinical data on the effectiveness and safety of repeating MR-HIFU (30, 42). Moreover, long-term outcomes of repeating MR-HIFU such as risk of pathological fracture, duration of pain response, retreatment rates are unknown in this early phase of implementation. The alternative of repeating MR-HIFU should be investigated in future models once further evidence becomes available.

The high populational EVPI (approximately EUR 180 Mio.) indicates that further studies would be worthwhile for reducing uncertainty (36). Moreover, the EVPPI enabled us to identify parameters that contribute most to decision uncertainty (i.e., MR-HIFU costs and fracture rates after MR-HIFU). An ongoing randomized controlled trial comparing MR-HIFU with either EBRT or a combination of both is currently recruiting patients with painful bone metastases (Clinical.trials.gov registration number NCT04307914). The results of this trial may clarify most of the uncertainty around patient relevant outcomes, especially the effectiveness in pain palliation.

In addition to the primary goal of pain palliation, a technology’s ability to induce local tumor control may contribute to the prevention of pathological fractures (5). Currently, data on local tumor control is based on stronger evidence for EBRT than for MR-HIFU. For instance, in our model, fracture rates for EBRT were taken from a large meta-analysis with 2468 patients (18), while the source of fracture rate for MR-HIFU was limited to an RCT with 112 patients (10), resulting in larger standard deviations for MR-HIFU and high EVPPI for MR-HIFU-related fracture rates. Preclinical evidence shows that MR-HIFU neither compromises the mechanical function of bones nor cause micro-cracks at the bone tissue level (43). However, improved evidence on fracture rates would be relevant for the cost-effectiveness of MR-HIFU and might be achieved by establishing prospective registries with the opportunity of embedded clinical trials.

Some limitations should be acknowledged. Firstly, choosing multi-fraction EBRT as the preferred comparator for our model may limit the generalizability of the results to other settings (44). The preference for single-fraction EBRT in many health systems may be justified by evidence on equivalent pain palliation and local tumor control, requirements to optimize machine availability and lower costs (44, 45). However, in Germany the fee-for-service reimbursement schemes seem to favor multi-fractionated schemes for radiotherapy practices (46), what in conjunction to physicians’ preferences, slows down the international trend toward hypo-fractionated schemes (47). Hence, our choice of comparator in the base case reflected EBRT practice in Germany (15, 16), and the recommendations from the German guideline on supportive therapies for oncologic patients (17). Additionally, in sensitivity analyses we explored the impact of different EBRT practices.

Secondly, costs with transportation to out-patient radiotherapy treatment are partially reimbursed but were excluded from our analysis due to lack of data. Nevertheless, transportation costs were expected to be very low (i.e., calculated as EUR 0.20 per km, and accounting for a fixed co-pay of EUR 5 to 10), hence the impact on model outputs are likely to be negligible. Thirdly, cancer patients can opt for rehabilitation after treatment with EBRT or MR-HIFU. Costs due to rehabilitation were not included in the analysis, because these costs are expected to incur in both groups. Moreover, costs with oncologic rehabilitation are commonly reimbursed by the German Pension Insurance.

Finally, although the clinical impact of adverse events on QALYs were accounted for, the corresponding costs associated with diagnosing and treating adverse events were not included. For example, 40% of patients treated with EBRT need symptomatic medication for nausea and vomiting for the first two weeks (19). However, the related costs (e.g., for anti-sickness tablets) are modest, and for MR-HIFU adverse events are reported in only 1% of the patients (10). Moreover, most adverse events related to MR-HIFU (e.g., discomfort or pain due to positioning, fatigue, numbness) are resolved prior to discharge with no need of additional diagnostic or treatment procedures (10). Hence, costs related to adverse events are unlikely to impact the model results.

Similar to our model, a previous cost-effectiveness Markov model from the US showed that MR-HIFU results in both additional costs and QALYs, yielding an ICER of $54,160 per QALY (29). However, because this model compared MR-HIFU with medication only, the comparability to our model results is limited. Our model is the first to compare a MR-HIFU-based strategy with EBRT, which is the current standard care. Moreover, the VOI analyses offers a refined information to decision-makers, highlighting the value of collecting more evidence on MR-HIFU to optimize health outcomes for patients with bone metastases.

## Conclusion

In summary, for patients with bone metastases the MR-HIFU-based strategy resulted in moderately higher costs and benefits in terms of both QALY (which accounted for adverse events of both treatments) and pain response compared to EBRT alone. Although there is still considerable uncertainty around the model results, this analysis can inform research prioritization, support decisions about reimbursement, and investments in infrastructure installation. Once further evidence is available, an updated economic modelling study would be opportune.

## Data availability statement

The original contributions presented in the study are included in the article/**Supplementary Material**. Further inquiries can be directed to the corresponding authors.

## Author contributions

Conceptualization and methodology, JG, SS, DM. Selection of input data: JG, SY, DA-B. Data imputation: JG. Verification: DM, AS. Face validation: DA-B, CB, HG, SY, CBo, HV. Analysis, JG, DM. Resources and funding acquisition: SS, HV. Writing – original draft preparation: JG, DM. Writing – review and editing, JG, SY, HG, GB, DA-B, CBa, CBo, HV, AS, SS, DM. Supervision and project administration: SS, DM. All authors contributed to the article and approved the submitted version.

## Funding

This project has received funding from the European Union’s horizon 2020 research and innovation programme under grant agreement No 825859.

## Conflict of interest

Author SY is employed part-time by Profound Medical GmbH.

The remaining authors declare that the research was conducted in the absence of any commercial or financial relationships that could be construed as a potential conflict of interest.

## Publisher’s note

All claims expressed in this article are solely those of the authors and do not necessarily represent those of their affiliated organizations, or those of the publisher, the editors and the reviewers. Any product that may be evaluated in this article, or claim that may be made by its manufacturer, is not guaranteed or endorsed by the publisher.
