# Supplementary material for: Early economic modeling of magnetic resonance image-guided high intensity focused ultrasound compared to radiotherapy for pain palliation of bone metastases
Source: Front Oncol. 2022 Sep 23;12:987546. doi: 10.3389/fonc.2022.987546 (PMC9537476; doi:10.3389/fonc.2022.987546)
Supplement: Supplementary file 1 [file DataSheet_1.docx]

Supplementary Material

Contents

**SM 1** Cost calculation of MR-HIFU and EBRT treatments based on diagnosis and procedure codes

**SM 2** Assessment of the Validation Status of Health Economic decision models

**SM 3** Value of Information Analysis (Methodological Supplement)

**SM 4** Deterministic sensitivity analysis (DSA) and structural sensitivity analysis results

**SM 5** Expected value of partial perfect information (EVPPI) results

**SM 1** **Cost calculation of MR-HIFU and EBRT treatments based on diagnosis and procedure codes**

Inpatient costs of EBRT were based on a 14-day length of stay (including one planning CT and consultation for informed consent (day one); irradiation simulation (day two), and irradiation on days nine to 14) (Table S1). Outpatient costs were calculated considering the treatment dose of 20Gy x5 and 8Gy x1 including the relevant surcharges (Table S2). Inpatient costs calculated for MR-HIFU are shown on table S3.

**Table S1.** Radiotherapy – outpatient codes and respective flat rates

|  | EbM code | Description | | | Flat rate |
| --- | --- | --- | --- | --- | --- |
| A | 25211 | Consultation flat rate for malignant disease or space-occupying processes of the central nervous system (once per treatment) | | | 117,28 € |
| B | 25214 | Consultation flat rate after radiotherapy treatment in accordance with the guidelines under the Ordinance on Protection against Damage Caused by Ionizing Radiation (Radiation Protection Ordinance) (once per treatment) | | | 28,95 € |
| C | 25342 | Computer-assisted radiation planning for percutaneous irradiation with individual dose planning for irregular fields with individual blocks, multi-lamella collimator, non-coplanar fields and/or 3-D planning | | | 473,18 € |
| D | 25343 | Surcharge for **fee schedule position** 25342 for computer-assisted high-precision radiation planning (IMRT and/or fractionated stereotaxy) (per radiation series) | | | 574,69 € |
| E | 34360 | CT-guided examination of organ sections for radiation planning in teletherapy or brachytherapy | | | 39,88 € |
| F | 01600 | **Medical report on the result of a patient examination** | | | 6,2 € |
| G | 01601 | **Physician's letter in form of individual written information from the physician to another physician about the patient's health or medical condition** | | | 12,17 € |
| H | 01602 | **Multiple Manufactures (e.g., copy) of a report or letter to the primary care physician** | | | 1,35 € |
| I | 40110 | **Flat-rate charge for sending or transporting a letter and/or written documents** | | | 0,81 € |
| J | 25321 | **Radiation with a linear accelerator for malignant diseases or space-occupying processes of the central nervous system** | | | 86,86 € |
| K | 25324 | **Surcharge for more than 1 target volume (malignant disease) [up to 2 each]** | | | 23,88 € |
| L | 25325 | Surcharge for high-precision technology for malignant diseases | | | 24,79 € |
| M | 25327 | **Surcharge for fee schedule position 25321 for irradiation using high-precision technology in combination with image-guided setting (IGRT)** | | | 47,32 € |
| N | 25328 | **Surcharge for fee schedule position 25321 if the individual dose is exceeded ≥ 2.5Gy** | | | 48,44 € |
| Total cost per patient: | | | | | |
| A+B+C+D+E+F+G+H+I+5x(J+K+L+M+N) | | | Cost per patient, 5x 4Gy | 2.410,96 € | |
| A+B+C+D+F+G+H+I+5x(J+K+L+M+N) | | | Cost per patient, retreatment (without CT simulation), 5x 4Gy | 2.371,08 € | |
| A+B+C+D+E+F+G+H+I+J+K+L+M+N | | | Cost per patient, 1x 8Gy | 1.485,80 € | |
| A+B+C+D+F+G+H+I+J+K+L+M+N | | | Cost per patient, retreatment (without CT simulation), 1x 8Gy | 1.445,92 € | |

**Table S2.** Radiotherapy – Inpatient codes and simulation for a 14-day length of stay (DRG 154A)

| Code | Description |
| --- | --- |
| C79.5 (ICD) | Secondary malignant neoplasm of bone and bone marrow |
| Z51.0 (ICD) | Radiotherapy session |
| 8-528.6 (OPS) | Irradiation simulation for external beam irradiation and brachytherapy: CT-guided simulation for external beam irradiation |
| 8-529.3 (OPS) | Radiation planning for percutaneous irradiation and brachytherapy: radiation planning for intensity modulated radiotherapy |
| 8-529.4 (OPS) | Radiation planning, fusion with CT and MRI |
| 8-527.2 (OPS) | Aid for fixation, complex |
| 3-990 (OPS) | Computer-aided image data analysis with 3D evaluation |
| 3-995 (OPS) | Dosimetry for therapy planning |
| 5x 8-520.0 (OPS) | Each irradiation, cave: Grouping each on 5 different days |
| 5x 8-522.d1 or 8-522.91 (alternative code) |  |
| Length of stay (14 days) | Total charges: 6409,71€ |
| Length of stay (10 days) | Total charges: 5864,15€ (without 8-528.6 (OPS)) |

**Table S3.** Magnetic Resonance Imaging-guided High Intensity Focused Ultrasound (MR-HIFU) – Inpatient codes and simulation for a 2-day length of stay

| Code | Description |
| --- | --- |
| C79.5 | Secondary malignant neoplasm of bone and bone marrow |
| 5-789.7 (OPS) | Other operations on bone: Destruction, by magnetic resonance guided focused ultrasound [MRgFUS] |
| 3-826 (OPS) | Magnetic resonance imaging of the musculoskeletal system with contrast material |
| 8-900 (OPS) | Intravenous anesthesia |
| Total charges: 3429.53 € | |

MRgFUS synonym for MR-HIFU

**SM 2** Assessment of the Validation Status of Health Economic decision models (AdViSHE)

The validation process of the model is reported below according to the questions of the AdViSHE checklist [1], which is divided in 4 parts:

**Part A: Validation of the conceptual model (2 questions)**

| A1/ Face validity testing (conceptual model): Have experts been asked to judge the appropriateness of the conceptual model? |
| --- |
| Experts with different backgrounds and expertise were asked to judge the appropriateness of the conceptual model. SYY, HG, CBos, HMV and GB have extensive expertise in MR-HIFU in Germany. BAB, CB are German radiation oncologists.  In this step, one main issue raised was that we had not considered in a first draft the single fraction EBRT (1x 8Gy). After discussion with experts, we decided to add a proportion of patients being treated with 1x 8Gy in the base case and sensitivity analysis. |

| A2/ Cross validity testing (conceptual model): Has this model been compared to other conceptual models found in the literature or clinical textbooks? |
| --- |
| The concept of the model was developed based in similar models comparing different strategies to the treatment of bone metastases. For instance, the health states considered are similar and the transitions and equivalent to other Markov models. The assumption regarding opioid intake (oxycodone) in all states except for complete pain relief was also applied in other models. |

**Part B: Input data validation (2 questions)**

| B1/ Face validity testing (input data): Have experts been asked to judge the appropriateness of the input data? |
| --- |
| All authors were asked to judge the clinical and effectiveness data.  In this step, the following issues were raised by experts:   1. Whether the retreatment rate applied to strategy A was appropriate.  - Due to lack of better data we kept the initial assumption. We tested a range of retreatment rates in sensitivity analyses.  1. If the data concerning EBRT practices and costs in Germany are possibly outdated, and that single fraction should be included.  - We corrected the base case according to expert opinion (adopting a more conservative approach of the proportion of outpatient EBRT– 70% instead of 60%- and also considering the single fraction EBRT as treatment alternative). |

| B2/ Model fit testing: When input parameters are based on regression models, have statistical tests been performed? |
| --- |
| We adjusted yearly values to fit the model’s monthly cycles and transformed rates into probabilities. These calculations were done by JSCG and reviewed by a second model expert (DM) and a statistician (AS). |

**Part C: Validation of the computerized model (4 questions)**

| C1/ External review: Has the computerized model been examined by modelling experts? |
| --- |
| Yes. The computerized model was checked by a modelling expert (DM), after data imputation by JSCG. |

| C2/ Extreme value testing: Has the model been run for specific, extreme sets of parameter values in order to detect any coding errors? |
| --- |
| To detect coding errors, we tested extreme values for cost data and utility data. Extreme values for proportions of outpatient EBRT and single fraction EBRT are reported in the supplementary material. |

| C3/ Testing of traces: Have patients been tracked through the model to determine whether its logic is correct? |
| --- |
| Yes, we reviewed individual trials to check for if the events occurring during patients’ lifetime were plausible. We used trackers (retreatment and fractures) to identify at cohort level if the model’s logic was correct. |

| C4/ Unit testing: Have individual sub-modules of the computerized model been tested? |
| --- |
| Yes. We tested and reported alternative scenarios (e.g. all patients undergoing MR-HIFU as first-line treatment, and all patients undergoing single fraction EBRT). |

**Part D: Operational validation (4 questions)**

| D1/ Face validity testing (model outcomes): Have experts been asked to judge the appropriateness of the model outcomes? |
| --- |
| Yes. The appropriateness of model outcomes was judged by all authors. |

| D2/ Cross validation testing (model outcomes): Have the model outcomes been compared to the outcomes of other models that address similar problems? |
| --- |
| Partially done, the model concept and data applied was similar to other models, but there was limited comparability with other models regarding results, since this is the first model to present the comparison MR-HIU vs. EBRT. |

| D3/ Validation against outcomes using alternative input data: Have the model outcomes been compared to the outcomes obtained when using alternative input data? |
| --- |
| Yes. We conducted several structural sensitivity analyses, reported in the main manuscript and in the supplementary material. |

| D4/ Validation against empirical data: Have the model outcomes been compared to empirical data? |
| --- |
| Not applicable. |

**SM 3** Value of Information (VOI) Analysis

The expected value of perfect information (EVPI) is the difference between the expected value of a decision made with perfect information and the value of the decision made with current evidence [2]. While the EVPI computes all input parameters simultaneously, the expected value of perfect partial information (EVPPI) quantifies how individual parameters or parameters sets contribute to decision uncertainty [2]. A hypothetical willingness-to-pay (WTP) of EUR 20.000/QALY was set to calculate the EVPI and EVPPI in terms of net monetary benefits (NMB). Initial estimates for WTP in high income countries suggest that thresholds should be set between 18-71% of the GDP per capita [3]. Hence, our assumption of EUR 20.000/QALY that is equivalent to approximately 50% of Germany’s GDP.

The PSA results were inputted to the Sheffield Accelerated Value of Information (SAVI), which uses regression-based methods to calculate the impact of parameter sets on decision uncertainty. For sets with up to five parameters, the GAM regression method is used. For subsets with five or more parameters the GP regression method is used [4].

Because MR-HIFU and radiotherapy are rapidly evolving technologies, the time horizon for the VOI analysis was defined at 5 years (i.e., the time in which the information would have most value). To calculate the populational EVPI, the per person EVPI was multiplied by the potential benefit population in Germany. The potential beneficial population (N) was calculated as:

$$N=P_{0}+\sum_{t=0}^{T} {\frac{I_{t}}{{(1+r)}^{t}}}$$

In that P_0_ = prevalent population at time t = 0, I_t_ = incident population at time t, r = discount rate (defined as 3%) [2].

The prevalence and incidence of stage IV breast cancer, stage IV prostate cancer, and stage IV lung cancer were taken from the German Centre for Cancer Registry Data [5], as shown in Table S5.

**Table S5.** Cancer specific prevalence and incidence rates applied to the calculation of the potential benefit population over 5 years

|  | 5-year prevalence | Incidence 2018 | Proportion Stage IV | Benefit Population over 5 years |
| --- | --- | --- | --- | --- |
| Breast cancer | 304,100 | 69,900 | 7% | 66,453 |
| Prostate Cancer | 260,400 | 65,200 | 18% | 112,355 |
| Lung cancer | 91,600 | 57,220 | 52% | 232,373 |
| Total |  |  |  | 411,181 |

**SM 4** Deterministic sensitivity analysis (DSA) results

**
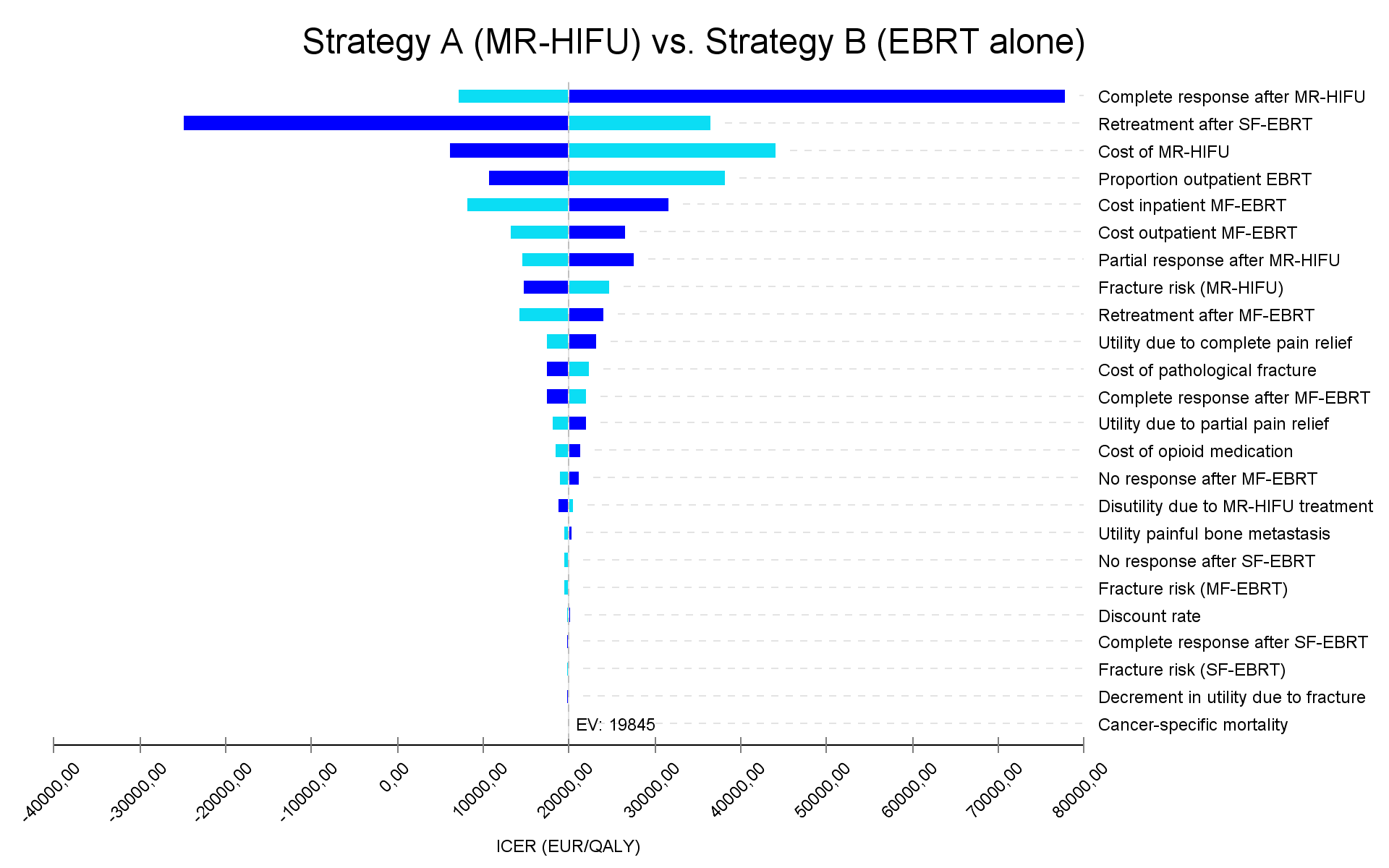
**

**Figure S1.** Deterministic sensitivity analyses for patients with bone metastases. The tornado diagrams show the results of deterministic sensitivity analyses (i.e., effect on the ICER by varying one model input parameter at a time, the higher values are represented by light blue bars, lower values by dark blue bars). Abbreviation: ICER: Incremental cost-effectiveness ratio.

**Table S6** Results from structural sensitivity analysis

| Proportion of 1x8Gy (at both strategies) | | | | | | |
| --- | --- | --- | --- | --- | --- | --- |
|  | Strategy | Cost (EUR) | Incremental Cost (EUR) | Effectiveness (QALY) | Incr.Effectiveness (QALY) | ICER (EUR/QALY) |
| 0% | Strategy B | 8,318 | - | 0.939 | - | - |
|  | Strategy A | 8,631 | 313 | 0.961 | 0.022 | 14,369 |
| 25% | Strategy B | 7,783 | - | 0.944 | - | - |
|  | Strategy A | 8,323 | 540 | 0.961 | 0.017 | 31,731 |
| 50% | Strategy B | 7,275 | - | 0.950 | - | - |
|  | Strategy A | 8,010 | 734 | 0.961 | 0.011 | 68,827 |
| 75% | Strategy B | 6,720 | - | 0.950 | - | - |
|  | Strategy A | 7,676 | 957 | 0.960 | 0.010 | 98,580 |
| 100% | Strategy B | 6,214 | - | 0.954 | - | - |
|  | Strategy A | 7,388 | 1174 | 0.961 | 0.01 | 168,392 |
| Proportion of outpatient EBRT (at both strategies) | | | | | | |
|  | Strategy | Cost (EUR) | Incremental Cost (EUR) | Effectiveness (QALY) | Incr.Effectiveness (QALY) | ICER (EUR/QALY) |
| 0% | Strategy B | 9,672 | - | 0.961 | - | - |
|  | Strategy A | 10,381 | 709 | 0.941 | -0.020 | dominated |
| 20% | Strategy B | 9,190 | - | 0.961 | - | - |
|  | Strategy A | 9,437 | 247 | 0.941 | -0.020 | dominated |
| 50% | Strategy A | 8,493 | - | 0.941 | - | - |
|  | Strategy B | 8,707 | 215 | 0.961 | 0.020 | 10,664 |
| 80% | Strategy A | 7,548 | - | 0.941 | - | - |
|  | Strategy B | 8,225 | 676 | 0.961 | 0.020 | 33,615 |
| 100% | Strategy A | 6,604 | - | 0.941 | - | - |
|  | Strategy B | 7,742 | 1138 | 0.961 | 0.020 | 56,566 |
| Retreatment rate (at strategy A) | | |  |  |  |  |
| Strategy | | Cost (EUR) | Incremental Cost (EUR) | Effectiveness (QALY) | Incr.Effectiveness (QALY) | ICER (EUR/QALY) |
| 8% | Strategy A | 8,115 | - | 0.941 | - | - |
|  | Strategy B | 8,500 | 385 | 0.961 | 0.021 | 18,531 |
| 16% | Strategy A | 8,115 | - | 0.941 | - | - |
|  | Strategy B | 9,181 | 1066 | 0.969 | 0.028 | 38,252 |
| 24% | Strategy A | 8,115 | - | 0.941 | - | - |
|  | Strategy B | 9,722 | 1607 | 0.978 | 0.037 | 43,095 |
| 32% | Strategy A | 8,115 | - | 0.941 | - | - |
| Cost-covering lump-sums MR-HIFU costs [6] | | | | |  |  |
| Mean:5147 | Strategy A | 8,115 |  | 0,937 |  |  |
|  | Strategy B | 9,663 | 1,548 | 0,957 | 0,020 | 77,650 |
| Lower:4092 | Strategy A | 8,115 |  | 0,937 |  |  |
|  | Strategy B | 8,958 | 843 | 0,957 | 0,020 | 42,253 |
| Upper:5876 | Strategy A | 8,115 |  | 0,937 |  |  |
|  | Strategy B | 10,151 | 2,036 | 0,957 | 0,020 | 102,109 |

**SM 5** Expected value of partial perfect information (EVPPI) results

**Figure S2.** Per person EVPPI for parameter sets. Abbreviations: EVPPI: Expected value of partial perfect information, MR-HIFU: Magnetic resonance-guided High intensity Focused Ultrasound, EBRT: External Beam radiotherapy, BM: Bone metastases, SKE: Skeletal-related events.

References

1. Vemer, P.; Corro Ramos, I.; van Voorn, G.A.; Al, M.J.; Feenstra, T.L. AdViSHE: A Validation-Assessment Tool of Health-Economic Models for Decision Makers and Model Users. *PharmacoEconomics* **2016**, *34*, 349-361, doi:10.1007/s40273-015-0327-2.

2. Fenwick, E.; Steuten, L.; Knies, S.; Ghabri, S.; Basu, A.; Murray, J.F.; Koffijberg, H.E.; Strong, M.; Sanders Schmidler, G.D.; Rothery, C. Value of Information Analysis for Research Decisions-An Introduction: Report 1 of the ISPOR Value of Information Analysis Emerging Good Practices Task Force. *Value in health : the journal of the International Society for Pharmacoeconomics and Outcomes Research* **2020**, *23*, 139-150, doi:10.1016/j.jval.2020.01.001.

3. Woods, B.; Revill, P.; Sculpher, M.; Claxton, K. Country-Level Cost-Effectiveness Thresholds: Initial Estimates and the Need for Further Research. *Value Health* **2016**, *19*, 929-935, doi:10.1016/j.jval.2016.02.017.

4. Strong, M.; Oakley, J.E.; Brennan, A. Estimating multiparameter partial expected value of perfect information from a probabilistic sensitivity analysis sample: a nonparametric regression approach. *Medical decision making : an international journal of the Society for Medical Decision Making* **2014**, *34*, 311-326, doi:10.1177/0272989x13505910.

5. Centre for Cancer Registry Data. Cancer in Germany Availabe online: <https://www.krebsdaten.de/> (accessed on 2nd May).

6. Simões Corrêa Galendi, J.; Yeo, S.Y.; Simic, D.; Grüll, H.; Stock, S.; Müller, D. A time-driven activity-based costing approach of magnetic resonance-guided high-intensity focused ultrasound for cancer-induced bone pain. *International journal of hyperthermia : the official journal of European Society for Hyperthermic Oncology, North American Hyperthermia Group* **2022**, *39*, 173-180, doi:10.1080/02656736.2021.2023768.
